# Barking up the wrong tree: Modern northern European dogs fail to explain their origin

**DOI:** 10.1186/1471-2148-8-71

**Published:** 2008-02-28

**Authors:** Helena Malmström, Carles Vilà, M Thomas P Gilbert, Jan Storå, Eske Willerslev, Gunilla Holmlund, Anders Götherström

**Affiliations:** 1 Department of Evolutionary Biology, Uppsala University, SE-752 36 Uppsala, Sweden; 2 National Board of Forensic Medicine, Department of Forensic Genetics and Forensic Toxicology, Linköping, Sweden; 3 Centre for Ancient Genetics, Biological Institute, University of Copenhagen, Denmark; 4 Osteoarchaeological Research Laboratory, Stockholm University, Stockholm, Sweden

## Abstract

**Background:**

Geographic distribution of the genetic diversity in domestic animals, particularly mitochondrial DNA, has often been used to infer centers of domestication. The underlying presumption is that phylogeographic patterns among domesticates were established during, or shortly after the domestication. Human activities are assumed not to have altered the haplogroup frequencies to any great extent. We studied this hypothesis by analyzing 24 mtDNA sequences in ancient Scandinavian dogs. Breeds originating in northern Europe are characterized by having a high frequency of mtDNA sequences belonging to a haplogroup rare in other populations (HgD). This has been suggested to indicate a possible origin of the haplogroup (perhaps even a separate domestication) in central or northern Europe.

**Results:**

The sequences observed in the ancient samples do not include the haplogroup indicative for northern European breeds (HgD). Instead, several of them correspond to haplogroups that are uncommon in the region today and that are supposed to have Asian origin.

**Conclusion:**

We find no evidence for local domestication. We conclude that interpretation of the processes responsible for current domestic haplogroup frequencies should be carried out with caution if based only on contemporary data. They do not only tell their own story, but also that of humans.

## Background

Mitochondrial phylogeography is a useful tool for the study of wild populations [1]. But applying phylogeography to domestic species is more complicated. For example, the arrival of dogs into the New World was not a simple expansion of Asian dog populations, but a consequence of human migrations [2]. Humans have a history of population movements and social changes over the last ten thousand years [3-6], and this, together with the human-mediated breeding history has affected the genetic composition of livestock animals [7]. Therefore, human influence cannot be neglected when studying patterns of genetic diversity in domesticates.

A classic example of the use of mitochondrial DNA (mtDNA) diversity to infer the history of domestication refers to dogs (*Canis familiaris*). Four to six mitochondrial haplogroups (Hg) have been described in genetic studies of modern dogs [8], indicating recurrent domestication or backcrosses between domestic dogs and wild wolves (*Canis lupus*). Three of the major Hgs are distributed throughout the world, whereas one (D) is restricted to Europe, especially in breeds originating in Scandinavia [8-11]. Similar patterns of fragmented genetic diversity have been used to argue for local domestication in other species [12]. Such scenario could apply to dogs as they appear as early as 9000 years ago in Scandinavia [13,14], and as dogs and wolf remains have been found on the same sites [15,16].

We have genetically typed mtDNA from ancient Scandinavian dog remains to investigate whether the high frequency of a rare dog Hg in northern Europe is evidence of a local domestication event. We predict that an early domestication event in Scandinavia would be visible in a notable frequency of Hg D in ancient Scandinavian dog remains.

## Results and Discussion

**Figure 1:**
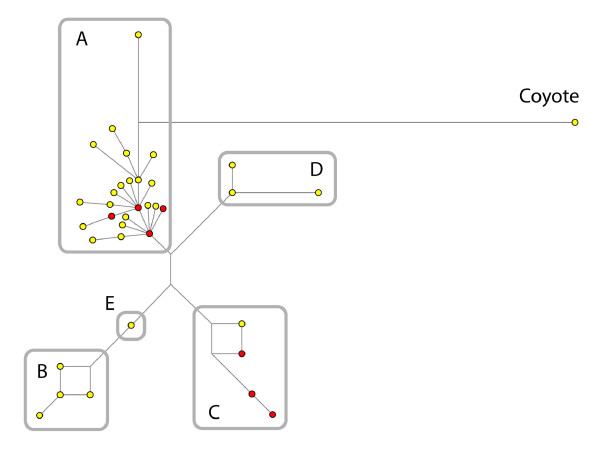
**Reduced median network based on 561 sequences (216 bp in length) including 18 ancient sequences (full sequences, not only those in Table 2) produced in this study and a coyote (*Canis latrans*) outgroup.** After removing tri-status characters, 36 haplotypes remain; those in red contain at least one ancient Scandinavian individual. A NJ tree based on Kimura-2 and bootstrapped with 1000 pseudoreplicates yielded a similar topology where Hg A was not supported, Hg B received 81% support, Hg C 84%, and Hg D 65%. Four medieval and three Neolithic dogs are distributed among the Hg A sequences, and 11 Neolithic sequences are distributed within the C clade.

Reproducible sequence data for the full 219 bp sequence was recovered from 18 samples. The remaining six specimens only yielded reproducible sequence data for a shorter 107 bp product (DQ860843-3 DQ860864, AY673648–AY673672, Table 1). Phylogenetic reconstruction including the 18 prehistoric dogs that yielded complete sequences and 543 modern dog sequences [11] revealed that all prehistoric dogs grouped within modern Hgs (Figure 1). Seven of the 18 sequences belonged to Hg A, the group that encompasses more than 70% of all tested modern dogs around the world [11], while the remaining eleven belonged to the now rare Hg C. Previously published data on 273 Swedish dogs indicated frequencies of the four major Hgs: A 69.2%, B 18.3%, C 7.0%, and D 5.5% [9]. When only native Scandinavian breeds were considered (n = 54), the haplotype frequency for Hg D increased to 33.3% [9]. A comparison of the 219 bp sequences obtained from the ancient dog specimens (n = 18) and the contemporary native breeds indicates that the frequency of Hg D is significantly reduced in the ancient data set (0% in the ancient dataset, 33% in the modern dataset, p < 0.001). The remaining six ancient Scandinavian dogs that yielded only 107 bp of reproducible sequence data, also indicated an absence of Hg D (Table 2). In total, eleven sequences had substitution patterns indicative for Hg A, while the remaining 13 had a substitution indicative for Hg C (Table 1).

**Table 1:** Description of material and Hg belonging.

Sample	Locality	Element	Age	Haplogroup
2^1,2^	Korsnäs^#^	Bone	Neolithic	C
3^1^	Korsnäs^#^	Bone	Neolithic	A*
4^1,2^	Korsnäs^#^	Bone	Neolithic	A
12^1,3^	Bergsgraven	Bone	Neolithic	C
13^1,2^	Ajvide	Bone	Neolithic	C
15^1,2^	Ajvide^#^	Bone	Neolithic	C
16^1,2^	Ajvide^#^	Bone	Neolithic	A
17^1,2^	Ajvide	Bone	Neolithic	C
18^1,2^	Ajvide	Bone	Neolithic	C
19^1,2^	Ajvide^#^	Bone	Neolithic	C
21^1,2^	Ajvide	Teeth	Neolithic	C
22^1,2^	Ajvide^#^	Teeth	Neolithic	C
23^1,2^	Ajvide	Teeth	Neolithic	C
25^1,3^	Ajvide	Tooth	Neolithic	C
26^1,3^	Skara A	Bone	Medieval	A*
27^1,2^	Skara B	Bone	Medieval	A
28^1^	Stockholm	Bone	Medieval	C*
30^2,3^	Visby	Teeth	Neolithic	A
32^3^	Eketorp	Bone	Medieval	C*
33^3^	Eketorp	Bone	Medieval	A*
34^3^	Eketorp	Bone	Medieval	A*
1243^3^	Sunnerby	Tooth	Medieval	A
229^3^	Sunnerby	Bone	Medieval	A
231^3^	Sunnerby	Bone	Medieval	A

Scandinavian dog remains yielding DNA. Sample name, locality, element (tooth or bone), age, and haplogroup are specified. Those independently replicated are marked with #, those only yielding the 107 bp fragment are marked with * (otherwise, the total length of the studied sequence is 219 bp). Sequences are from Malmström et al 2005^1 ^(107 bp), Malmström et al 2007^2 ^(112 bp), and from this study^3 ^(either or both fragments).

**Table 2:** Description of genetic data.

	*15595*	*15611*	*15625*	*15626*	*15638*	*15639*	*15650*	*15652*	*15665*	*Hg*
**Ref**	**C**	**T**	**T**	**A**	**T**	**T**	**T**	**G**	**T**	**-**
2	·	·	·	·	G	·	C	·	·	C
3	·	·	·	·	A	·	·	·	C	A
4	·	·	·	G	A	·	·	·	C	A
12	·	·	·	·	G	·	C	·	·	C
13	·	C	·	·	G	·	C	·	·	C
15	·	C	·	·	G	·	C	·	·	C
16	·	·	·	·	A	·	·	·	C	A
17	·	C	·	·	G	·	C	·	·	C
18	·	C	·	·	G	·	C	·	·	C
19	·	C	·	·	G	·	C	·	·	C
21	·	C	·	·	G	·	C	·	·	C
22	·	C	·	·	G	·	C	·	·	C
23	·	C	·	·	G	·	C	·	·	C
25	·	C	·	·	G	·	C	·	·	C
26	·	·	·	G	A	·	·	·	·	A
27	·	·	·	G	A	·	·	·	·	A
28	·	C	·	·	G	·	C	·	·	C
30	·	·	·	·	A	·	·	·	C	A
32	·	C	·	·	G	·	C	·	·	C
33	·	·	·	G	A	·	·	·	·	A
34	·	·	·	·	A	·	·	·	·	A
1243	·	·	·	·	·	·	·	·	·	A
229	·	·	·	·	·	·	·	·	·	A
231	·	·	·	·	·	·	·	·	·	A

Haplogroup-specific substitutions in a 107 bp fragment of the D-loop (15561–15668) and their occurrence in 24 ancient Scandinavian dogs. The reference sequence is from Kim *et al*. 1998.

When the complete ancient dataset was compared to the contemporary dataset, it proved equally improbable (p < 0.001) that Hg D was present in the oldest specimens in a frequency similar to that observed in modern dogs. Using a binomial distribution, we estimate that the frequency of Hg D must have been lower than 0.118 in the original population for us not to have noticed it in 24 samples (p < 0.05), and lower than 0. 182 during the Neolithic for us not to have noticed it in 15 samples (p < 0.05). Lastly, the χ^2 ^test between the Hg frequencies of the complete ancient dataset with that of the complete Scandinavian (not only considering native breeds) demonstrated a significant deviation between the modern and ancient frequencies (p < 0.0388, χ^2 ^= 4.27, df = 1).

Our results indicate that Hg frequencies have been altered in Scandinavian dogs since their first arrival. Interestingly, while Hg C is overrepresented in our ancient material, there is a complete lack of the Scandinavian group D in our ancient dataset. Hg D is the one that could support a Scandinavian origin whereas Hg C is suggested to be of Asian origin [11]. Thus, we find no obvious evidence for prehistoric canid domestication in Scandinavia. An external origin of Scandinavian dogs is supported by morphologic data, as even the oldest remains of dogs in Scandinavia were of smaller size than those of prehistoric and extant wolves [15,17]. While canid domestication may have occurred in other parts of Europe [18], Scandinavian dogs were likely imported and had experienced a long period of morphological change under human control before they reached the Scandinavian peninsula.

## Conclusion

In a wider context, our data calls for caution when using modern sequences of domestic animals to interpret the history of domestication. There is a profound difference on selective forces on wild and domestic animals. Domesticates are subject to constant manipulation and follow the same historic vicissitudes as the associated human population. For example, genetic diversity may change rapidly due to intense selective breeding, backcrossing with the wild ancestor [19-22], migration of human groups with genetically different domesticates [2], and decimation of local populations by invaders [23]. Hg frequency could change over a short period of few generations. While diversity and branching pattern of mitochondrial DNA may be used to study the number and possible antiquity of domestication events, phylogeographical patterns in domestic species may indeed not be suitable to base discussion on their geographical origin. Close collaboration between zooarchaeologists and molecular biologists, involving the study of ancient specimens, may be the only way to verify the continuity through time of the species that have set the basis of our culture.

## Methods

**Figure 2:**
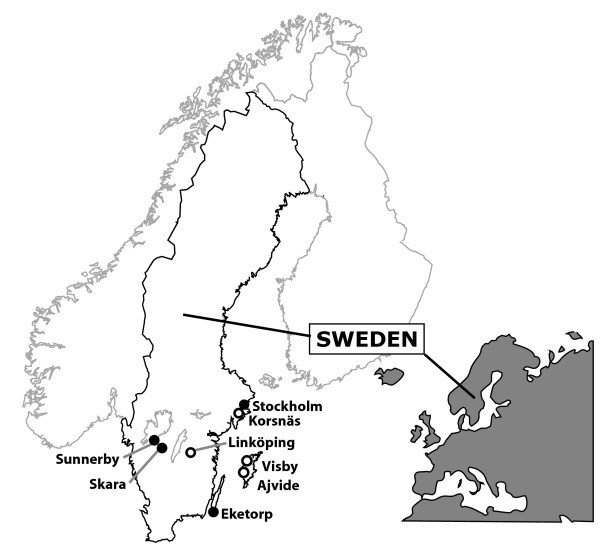
Samples were collected from eight different archaeological localities in southern Scandinavia, four Neolithic (empty circles) and four medieval (black dots).

We analysed sequences from 24 archaeological domestic dogs. The skeletal material represents eight Scandinavian sites: four Neolithic (≈5300–4500 BP) and four Medieval (≈1000–500 BP) (Table 1, Figure 2). DNA was extracted, PCR-amplified and sequenced according to previously published protocols [24,25] (Table 1). A subset of the sample sequences (n = 7) was replicated in an independent laboratory (Center for Ancient Genetics, University of Copenhagen) confirming the original results in all cases. The new data was combined with previously published data [24,25] for analysis (see Table 1). Most of the Neolithic samples, those from Ajvide, Visby, and Korsnäs (Figure 2), were recovered as isolated findings in cultural layers. One sample was from a complete skeleton found in a burial in Central Sweden (Bergsgraven in Linköping). The Medieval samples originated from isolated skeletal remains recovered in urban (Skara and Stockholm) or rural contexts (Eketorp, Sunnerby).

We used polymorphisms found within a 219 bp fragment of the dog D-loop region and comparison with previously published sequences to determine Hgs. To portray the relationship between sequences we constructed a reduced median network, including all 219 bp, using Network 4.1 [26]; threshold was set to 1. The default parameters for reduction were used, where parallel mutations are assumed to be more frequent between existing sequences, than between an existing sequence and a median vector. As the reduction was intense, a NJ tree was constructed to support the network using the Kimura 2-parameter model of sequence evolution. Support for the nodes was assessed with 1000 bootstrap pseudoreplicates.

The Hg frequencies within the ancient datasets were compared to those in modern Scandinavian dogs [9] using χ^2 ^tests. To ensure a minimum sample of 5 for all cells in χ^2 ^tests, we pooled Hgs A and D, and C and B (following the best natural grouping according to the network, Figure 1). Finally, we used the binomial distribution to estimate the probability of observing as many sequences from Hg D as found in the ancient samples assuming that the relative frequency was the same as in modern native Scandinavian dogs.

## Authors' contributions

HM did all experimental work apart from the independent replications, and helped analyse the data and write the paper. CV did part of the analytical work, and helped write the paper. TG and EW did the independent replication. TG revised the language. JS did the morphological selection, and described the archaeology. GH supervised the experimental work. AG did the analytical work together with HM and CV, and wrote the paper together with HM and CV. All authors read and approved the final manuscript.
